# EEG brain oscillations are modulated by interoception in response to a synchronized motor vs. cognitive task

**DOI:** 10.3389/fnana.2022.991522

**Published:** 2022-09-23

**Authors:** Laura Angioletti, Michela Balconi

**Affiliations:** ^1^International Research Center for Cognitive Applied Neuroscience (IrcCAN), Università Cattolica del Sacro Cuore, Milan, Italy; ^2^Research Unit in Affective and Social Neuroscience, Department of Psychology, Università Cattolica del Sacro Cuore, Milan, Italy

**Keywords:** interoceptive attentiveness, synchronization task, EEG, motor task, cognitive task

## Abstract

So far, little is known about how conscious attention to internal body signals, that is, interoception, affects the synchronization with another person, a necessary or required social process that promotes affiliations and cooperation during daily joint social interactions. The effect of explicit interoceptive attentiveness (IA) modulation, conceived as the focus on the breath for a given time interval, on electrophysiological (EEG) correlates during an interpersonal motor task compared with a cognitive synchronization task was investigated in this study. A total of 28 healthy participants performed a motor and a cognitive synchronization task during the focus and no-focus breath conditions. During the tasks, frequency bands (delta, theta, alpha, and beta bands) from the frontal, temporo-central, and parieto-occipital regions of interest (ROIs) were acquired. According to the results, significantly higher delta and theta power were found in the focus condition in the frontal ROI during the execution of the motor than the cognitive synchronization task. Moreover, in the same experimental condition, delta and beta band power increased in the temporo-central ROI. The current study suggested two main patterns of frequency band modulation during the execution of a motor compared with the cognitive synchronization task while a person is focusing the attention on one's breath. This study can be considered as the first attempt to classify the different effects of interoceptive manipulation on motor and cognitive synchronization tasks using neurophysiological measures.

## Introduction

The “concentrated attention to a particular interoceptive signal during a predetermined time interval” is known as interoceptive attentiveness (IA) (Schulz, 2016; Tsakiris and De Preester, 2018). As a top-down process requiring focused attention specifically on the breath, IA was originally thought to be the basis of mindfulness-based practices, controlled breathing, or even brief relaxation techniques (Farb et al., 2013; Weng et al., 2021). Indeed, the consciously focused attention on breath that underlies these practices has been shown to enhance a number of cognitive and emotional processes, such as the regulation of emotions (in terms of reduction in the negative affect; Arch and Craske, 2006), sustained attention, cognitive monitoring, and meta-awareness, as examples of cognitive processes based on interoceptive inputs (Weng et al., 2021), the observation of pain empathic reaction (Balconi and Angioletti, 2021b), and stress regulation (Grossman, 2011). However, nowadays, there are limited studies on the effect of IA on synchronization processes.

Only recently, research on interoception started focusing on the link between the perception of inner signals derived from within the body and their link with social processes (Arnold et al., 2019). In particular, the impact of interception on social synchronization—a necessary or essential social activity that encourages affinities and cooperation during routine joint social interactions—has been little investigated from a neurofunctional perspective.

In the neuroscientific literature, the most common behavioral synchrony tasks used to study synchronization are those involving movement or language (for a review see Balconi and Vanutelli, 2017). Former studies looked at the electrophysiological (EEG) brain correlates of synchronization during motor and linguistic imitation tasks. Notably, the hyperscanning paradigm advent in neuroscience (Montague et al., 2002; Balconi and Vanutelli, 2017) allowed deepening the neurophysiological mechanisms (inter-brain coupling) that guide interpersonal synchronization.

For example, during joint tapping motor tasks, the prefrontal regions of two interacting agents showed synchronization (Funane et al., 2011; Cui et al., 2012; Holper et al., 2012; Cheng et al., 2015; Baker et al., 2016; Pan et al., 2017). It was previously observed that guitarist pairs demonstrated more synchronized theta and delta oscillations in frontal and central electrode sites when playing a brief melody: this may be due to coordinated firing of neuronal assemblies in the motor and somatosensory cortex, which regulate and orchestrate motor activity, as well as frontal regions supporting social cognition (Lindenberger et al., 2009).

Through a leader–follower hand movement task, Yun et al. (2012) demonstrated the occurrence of subconscious movement synchronicity when engaging with another individual, and that theta and beta frequency bands across the inferior frontal gyrus, anterior cingulate, parahippocampal gyrus, and post-central gyrus displayed greater phase synchronization after the imitation phase.

Similarly, Dumas et al. (2010) instructed participants to mimic the other's hand movement while using a video feedback system. The right centro-parietal regions of the two brains showed stronger inter-brain phase synchronization in the mu, beta, and gamma range during behavioral synchrony.

In addition, the hyperscanning paradigm has been applied to investigate live interactive speech, thus enhancing neuroscientific accounts of live verbal interaction and social interaction (Jiang et al., 2012; Scholkmann et al., 2013; Liu et al., 2017; Hirsch et al., 2018; Zhang et al., 2018; Descorbeth et al., 2020). In a recent systematic review of hyperscanning studies on spoken communication and language (Kelsen et al., 2022), it was documented how brain synchrony primarily engages the frontal and temporo-parietal areas, which underlie the mirroring and mentalizing mechanisms that are active during communication dynamics. Specifically for EEG frequency bands, theta/alpha oscillatory amplitudes were found to be enhanced and synchronized between two subjects in the same bilateral temporal and lateral parietal regions during a human–human alternating speech task (Kawasaki et al., 2013). Also, alpha coherence was the highest in dyads that engaged in mutual eye gaze before class discussions compared to other dyads (Dikker et al., 2017). To go more in-depth in terms of neuroanatomical localization, Pérez et al. (2017) reported that neural alignment regarding alpha band wave activity was detected for listeners in the frontal region and speakers in the central region, and with respect to the theta band, in the temporal region for the listener and the frontal region for the speaker.

However, taken together, the hyperscanning works described previously did not manipulate IA (intended as the deliberate attention on the breath) during the synchronization tasks. To the best of our knowledge, the literature that delves into the effects of IA on single brain neural correlates during a motor or cognitive synchronization task is still scarce. To better understand this phenomenon at the cortical level on a single individual, a previous study explored the hemodynamic changes related to the effects of a brief focus on the breath session on a single brain.

Indeed, in a recent pilot study, the hemodynamic correlates of simple tasks requiring cognitive (linguistic) or motor synchronization were examined in order to determine the impact of the focus on the breath on such tasks. According to the results, adjusting the attention to the breath might broaden the benefits of boosting the brain PFC response to synchronization during basic synchronization tasks. Furthermore, it was shown that when the intentional focus on the breath was achieved during the cognitive task requiring synchronization with a partner, brain areas linked with sustained attention, such as the right PFC, were more involved. It is interesting to note that this evidence was significantly observed for the cognitive (i.e., linguistic) task, and not for the motor task. If taking into account the direct correlation between interoceptive correlates (and the focus placed on controlling them) and motor performance, as well as the neuroanatomic closeness between the interoceptive and the motor region, this finding appeared paradoxical (Balconi and Angioletti, 2022). Furthermore, this pilot study considered a limited sample size, and the functional near-infrared spectroscopy (fNIRS) technique was not applied to the somatosensory cortical regions or the rest of the brain, but simply over the PFC (Balconi and Molteni, 2016).

Other research has revealed that neuroimaging and EEG markers of cognitive control co-vary across mind-wandering (MW) occurrences, MW awareness, and the return to concentrate on the breath while performing a breath monitoring task (Braboszcz and Delorme, 2011; van Son et al., 2019). Recently, there has been considerable interest in spectrally characterizing these changes during focused attentional activities (Baldwin et al., 2017; Compton et al., 2019; Arnau et al., 2020), with delta, theta, alpha, and beta frequency bands all varying with the incidence of MW.

EEG is a low-cost, convenient method for recording brain electrical signals with high temporal precision at millisecond intervals. The minimal auditory noise produced by EEG equipment (as opposed to that from magnetic resonance scanners) makes it simpler for people to converse in an ecological and naturalistic continuous stream. Despite the low spatial resolution of EEG limits the degree to which specific areas of neural activity can be precisely localized, it nonetheless provides an indication of regional scalp activation.

Thus, we believe it could be of interest to investigate the EEG cortical effects of the focus on the breath (in terms of EEG frequency bands: delta, theta, alpha, and beta) in relation to distinct regions of interest while a person is performing a motor compared to a cognitive task requiring synchronization with a partner. For these reasons, in the present study, we propose the concomitant execution of the focus on the breath task and a motor or cognitive synchronization task, to test the effect of explicit IA manipulation on EEG correlates of a task requiring synchronization.

On the basis of the research and data described before, it was proposed that the frequency bands associated with sustained attention and attention control during interoceptive tasks (alpha, theta, and beta; Lomas et al., 2015; Villena-González et al., 2017; Colgan et al., 2019) will increase when inducing an explicit IA focus on the breath compared with the control condition during the synchronization tasks.

Moreover, given the positive effect of the focus on the breath condition over cognitive functioning, we do also expect that the frequency bands associated with the synchronization tasks could be enhanced in the explicit IA condition. In particular, during the execution of the motor synchronization task in the focus condition, we expect to observe theta and delta oscillations in frontal and central electrode sites, which may indicate coordinated and controlled motor activity (Lindenberger et al., 2009) and the increase of beta bands in the posterior regions during behavioral synchrony.

In line with Kawasaki et al. (2013) evidence, we suppose to find the presence of mainly alpha and theta band wave activity in the temporal and parietal regions during the cognitive synchronization task performance (i.e., a simple linguistic task). This pattern is supposed to be specifically enhanced in the focus on the breath compared to the control condition.

## Methods

### Participants

A total of 28 healthy individuals [22 women and six men; age mean (M) = 24.2 years; standard deviation (SD) = 3.11] were involved in the study by employing a non-probabilistic convenience sampling method. Exclusion criteria for participation in the study encompassed physiological conditions such as chronic or acute pain, severe medical and chronic diseases, seizures, traumatic brain damage, pregnancy, prior meditation experience, and any mental or neurologic abnormalities. All people who took part in the experiment were right-handed and had a normal or corrected-to-normal vision. All participants signed a written informed consent form before the experiment and were informed they would not be remunerated. The approval for this study was provided by the Ethical Committee of the Department of Psychology at the Catholic University of the Sacred Heart of Milan (protocol number: 2020TD), in accordance with the Declaration of Helsinki (1964).

### Procedural steps

The participants were situated in a dimly lit room with a researcher in charge of providing the experimental instructions and completing the synchronization tasks. Before beginning the experimental activities, EEG was used to record a 120-s resting baseline.

The subjects were asked to perform two simple motor and cognitive synchronization tasks by imitating the experimenter while their electrophysiological activity was recorded continuously. In this version of the alternating speech task, the participants were asked to pronounce four syllables “LA,” “BA,” “CA,” and “DA” sequentially and alternately. Each linguistic synchronization task session lasted about 3 mins, consecutively. Instead, the motor synchronization task consisted of finger movement. Specifically, the participants had to synchronize their finger movements with the experimenter sitting in front of them. The total duration of the task was of 3 mins (for the full description of the tasks; see Balconi and Angioletti, 2022).

The order of task execution was randomized and counterbalanced to prevent potential biases due to sequence effects. Each participant completed two basic synchronization tasks in the same day, under two different conditions: explicit IA and control conditions (Balconi and Angioletti, 2021a,b). The participants in the focus (explicit IA) condition were advised to focus on their breath while executing the activity, as follows: “*During this task, we ask you to concentrate on your breathing. Try to pay attention to how you feel and whether your breathing changes as you complete the activity.”* Instead, the participants in the no-focus condition received no request to focus on their interoceptive correlates.

**Figure 1:**
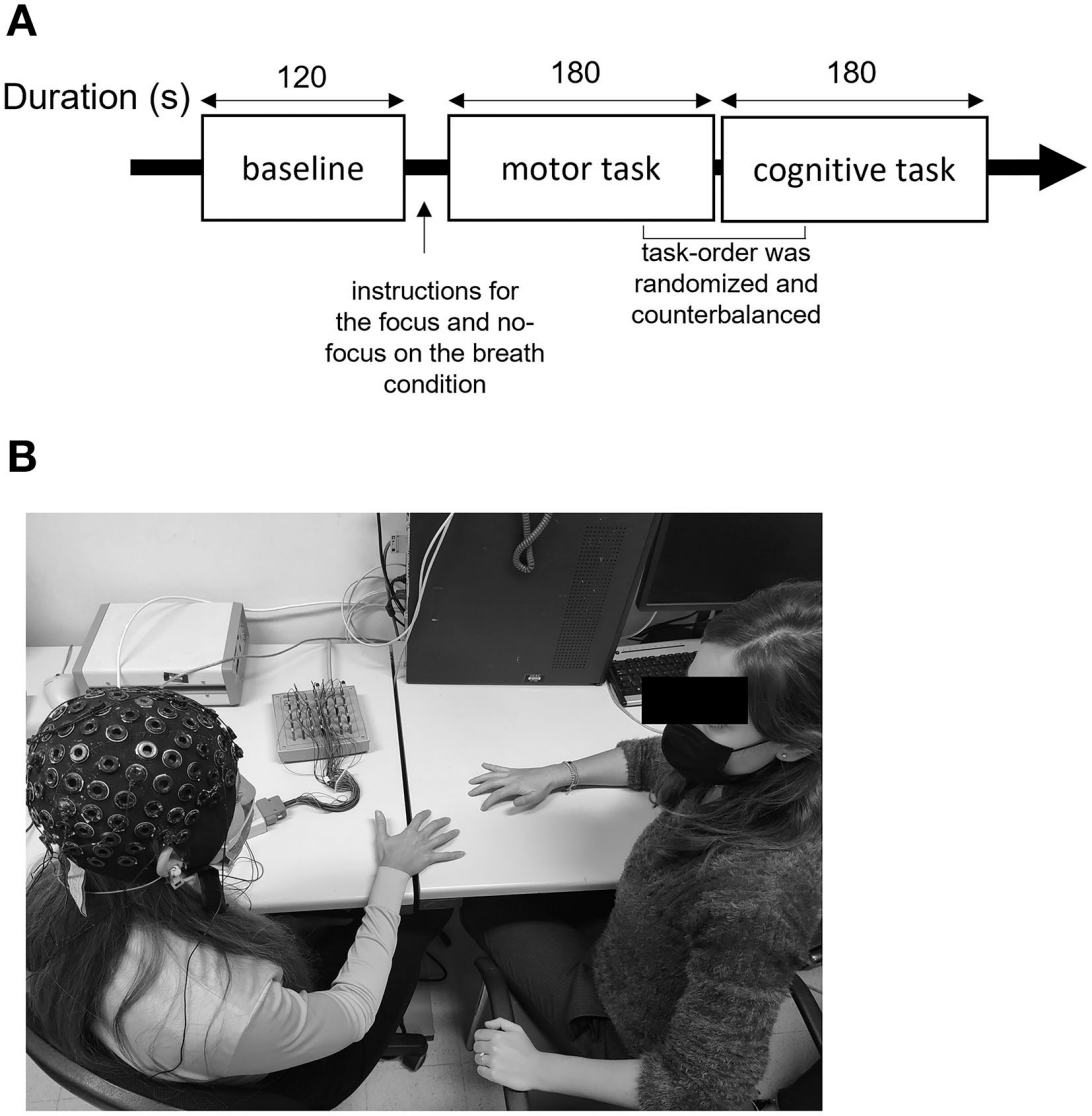
Description of the experiment setting. **(A)** Description of the procedure including baseline, motor, and cognitive synchronization tasks with and without focus on the breath manipulation. **(B)** Experimental setup, where the researcher performs the motor synchronization task and EEG equipment is used to collect the data.

To avoid biases brought on by sequence effects, the order in which the tasks were completed was randomized and counterbalanced. The entire experiment lasted <30 mins (Figure 1).

### EEG recording and signal reduction

A 32-channel amplifier (SynAmps system) and acquisition software (Neuroscan 4.2) were used to collect EEG data during task execution. An ElectroCap with Ag/AgCl electrodes was used to record EEG from active scalp sites referred to earlobes (10/20 International system of electrode placement) (Jasper, 1958). The EEG montage included the following 15 electrodes: Fp1, Fp2, AFF5h, Fz, AFF6h, T7, C3, Cz, C4, T8, P3, Pz, P4, O1, and O2. In addition, two EOG electrodes were placed on the outer canthi to detect eye movements. Prior to data collection, each subject's electrode impedance was measured and kept under 5 kΩ. Data were collected at a sampling rate of 500 Hz and then offline-filtered with a 0.01- to 30-Hz IIR bandpass filter (slope: 48 dB/octave). After that, the data were divided and examined visually for ocular, muscular, and movement artifacts. To compute the average power spectra, the fast Fourier transform (Hamming window, resolution: 0.5 Hz) was performed on artifact-free segments. The normalization-applied procedure consisted in the baseline (as the 120-s resting baseline was recorded at the start of the experiment) correction of the signal, by using the z-score transform. The successive comparisons were made by using the normalized values; 1,000-ms segments were subjected to the fast Fourier transform and successively averaged for each condition and each channel. The average power spectrum was then calculated for the major EEG frequency bands (delta 0.5–3.5 Hz, theta 4–7.5 Hz, alpha 8–12.5 Hz, and beta 13–30 Hz). Before the tasks, a 120-s resting baseline was recorded at the start of the experiment. The EEG biosignal was processed by Analyzer 2.0 software (Brain Products GmbH, Gilching, Germany).

**Figure 2:**
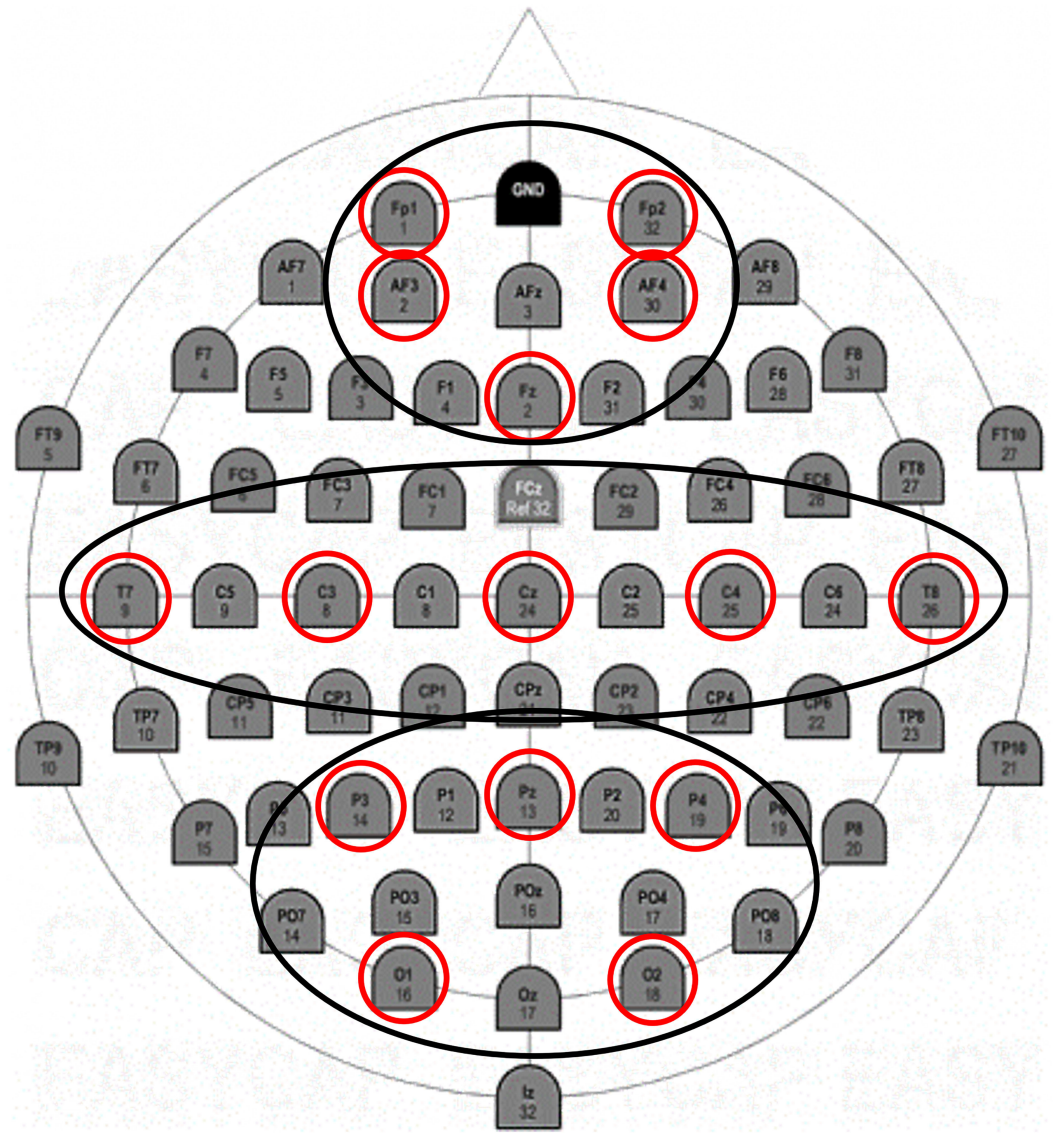
EEG montage. Positioning of 15 electrodes according to the 10–20 international system.

In the statistical analysis of the data, three regions of interest (ROIs) grouping by averaging frontal (F: Fp1; Fp2; AFF5h; AFF6h), temporo-central (TC: T7; T8; C3; C4), and parieto-occipital (PO: P3; P4; O1; O2) electrodes were considered (Figure 2).

### Statistical analysis

A total of four repeated-measures ANOVA with independent within-factors Task (2: motor and cognitive) × Condition (2: focus and no-focus) × *ROI* (3: frontal, temporo-central, and parieto-occipital) were applied to dependent EEG data (delta, theta, alpha, and beta power). In the case of significant interactions, pairwise comparisons were utilized to explore the significant interactions, and Bonferroni correction was applied to decrease potential biases in repeated comparisons. When applicable, Greenhouse–Geisser epsilon was used to correct the degrees of freedom in all ANOVA testing. The kurtosis and asymmetry indices were also employed to assess the data distribution normality. The size of statistically significant effects was evaluated through partial eta-squared (η^2^) indices. The statistical tests were performed with IBM SPSS Statistics version 25.

## Results

**Figure 3:**
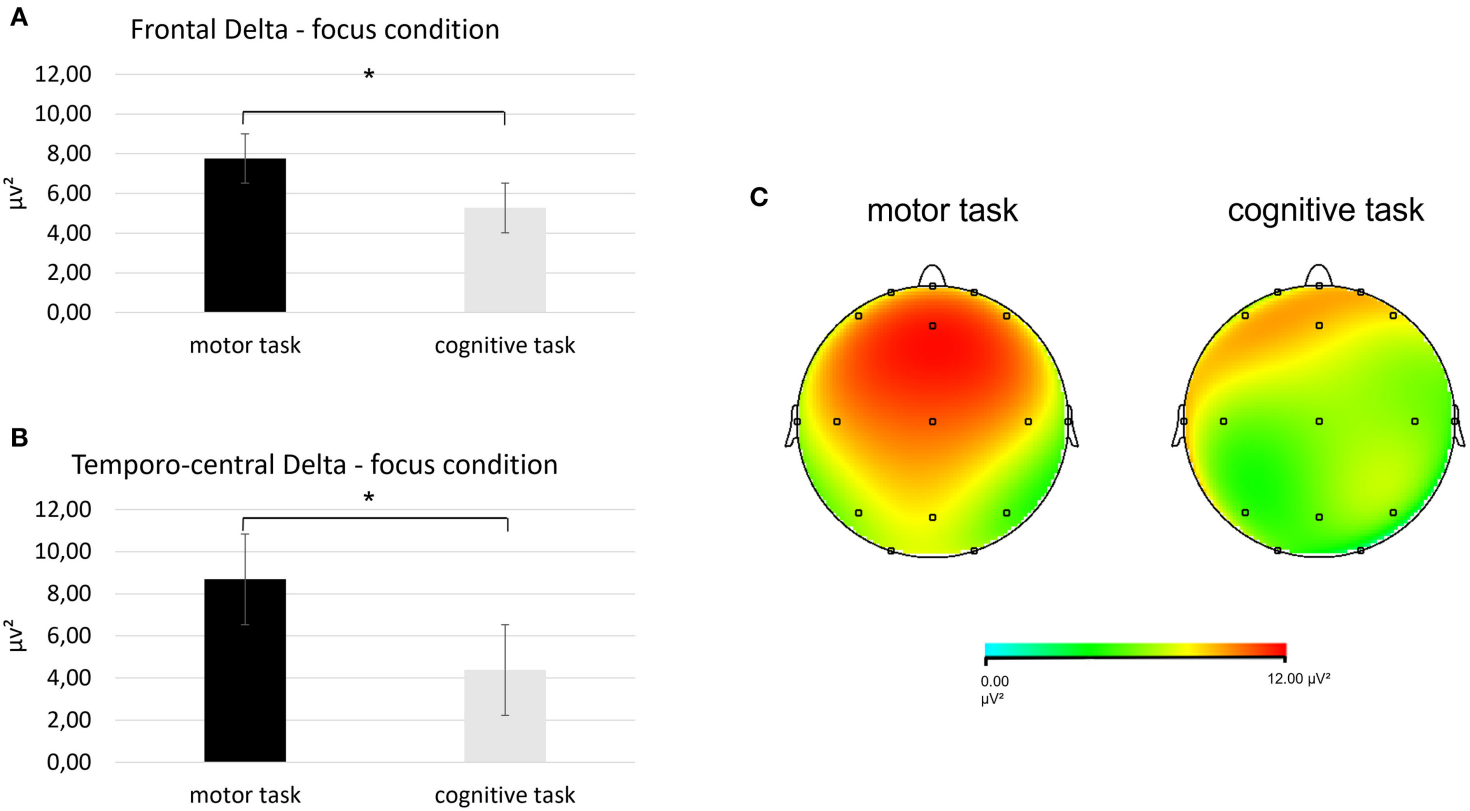
Results for EEG delta band. **(A)** Bar chart showing frontal delta mean values in the focus condition during the two synchronization tasks. **(B)** Bar graph displaying the significant increase in temporo-central delta values in the focus condition when performing the two synchronization tasks. **(C)** Red area represents greater delta power in the focus condition during the motor (left head) than the cognitive task (left head). For all charts, bars indicate ±1 standard error (SE); all asterisks mark statistically significant differences, with *p* ≤ 0.05.

First, regarding the delta band, a significant interaction effect *Task* × *Condition* × *ROI* was found [*F*_(2, 27)_ = 6.78, *p* < 0.001, η^2^ = 0.34]. Pairwise comparisons revealed greater mean delta values in the frontal ROI during the focus condition in the motor than in the cognitive task [*F*_(1, 27)_ = 7.03, *p* < 0.001, η^2^ = 0.409]. Also, higher mean delta values were found in the temporo-central ROI during the focus condition for the motor than in the cognitive task [*F*_(1, 27)_ = 7.62, *p* < 0.001, η^2^ =0.432] (Figures 3A–C).

**Figure 4:**
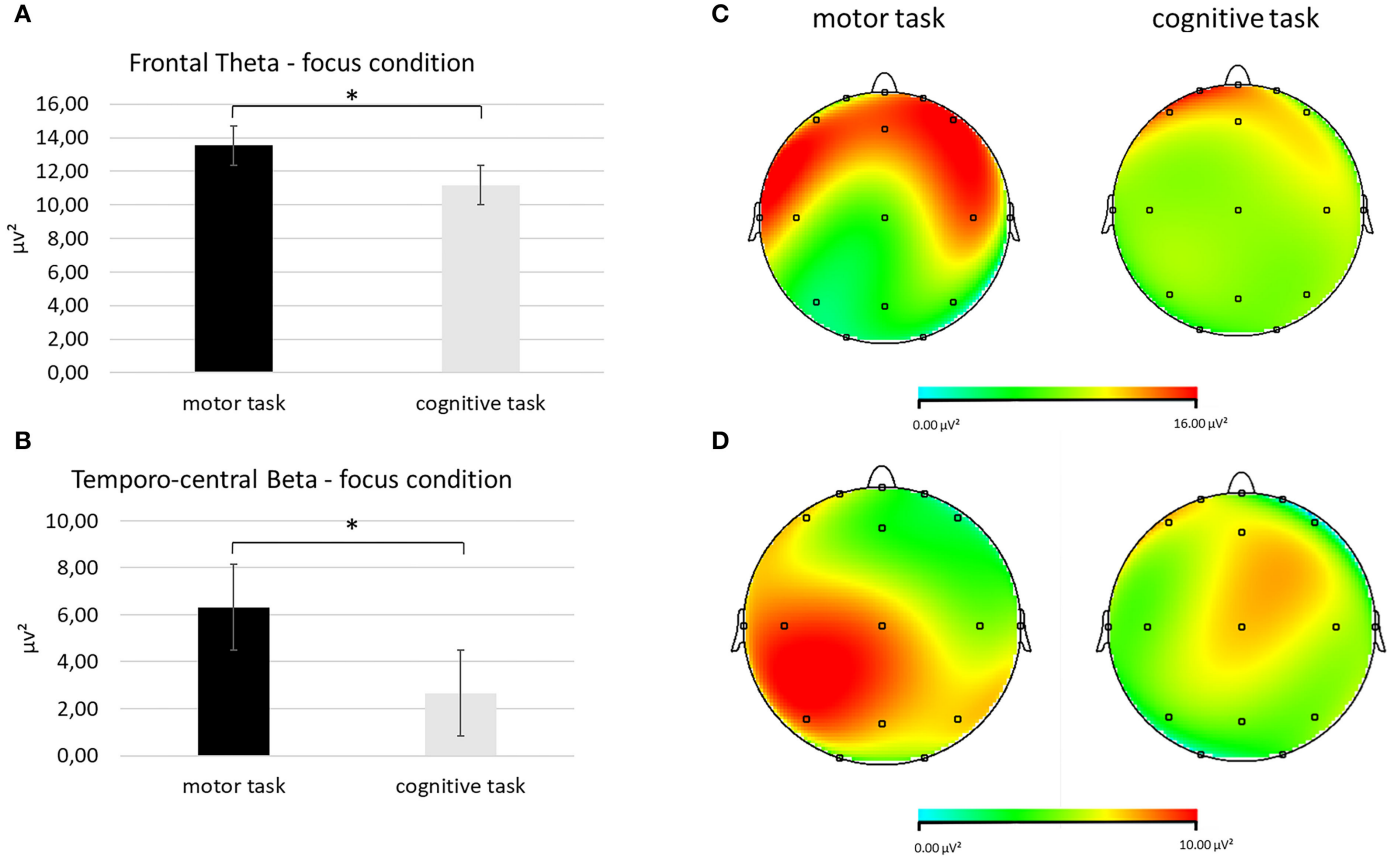
EEG theta and beta band findings. **(A)** Bar chart showing frontal theta mean values in the focus condition during the two synchronization tasks. **(B)** Topographical mapping representing the theta power enhancement in the frontal regions (red area) in the focus condition during the motor compared with the cognitive synchronization task. **(C)** Bar graph displaying the significant increase in temporo-central beta values in the focus condition when performing the two synchronization tasks. **(D)** Red area represents higher beta power in the focus condition during the motor (left head) than the cognitive task (left head). For all charts, bars indicate ±1 standard error (SE); all asterisks mark statistically significant differences, with *p* ≤ 0.05.

Second, for the theta band, it was detected a significant interaction effect *Task* × *Condition* × ROI [*F*_(2, 27)_ = 8.09, *p* < 0.001, η^2^ = 0.476]. Pairwise comparisons showed greater mean theta values in the frontal ROI during the focus condition in the motor than in the cognitive task [*F*_(1, 27)_ = 7.76, *p* < 0.001, η^2^ = 0.457] (Figures 4A,B).

A third significant interaction effect *Task* × Condition × ROI was identified for the beta band [*F*_(2, 27)_ = 8.90, *p* < 0.001, η^2^ = 0.490]. Higher mean values of the beta band were found in the temporo-central ROI during the focus condition in the motor than in the cognitive task [*F*_(1, 27)_ = 9.06, *p* < 0.001, η^2^ = 0.498], as shown by pairwise comparisons (Figures 4C,D).

No significant effects were found for the alpha band, and no other significant effects were found. Analyzer 2.0 (Brain Products GmbH, Munich, Germany) software was adopted for EEG data visualization.

## Discussion

The current work is the first to describe the effects of an explicit IA manipulation (conceived as a brief breath awareness practice) on the EEG correlates in response to a motor and a cognitive synchronization task. In particular, the EEG frequency bands (delta, theta, alpha, and beta) were observed and analyzed considering three main ROIs grouping frontal, temporo-central, and parieto-occipital electrodes. The results showed significantly higher delta and theta power in the focus condition in the frontal ROI during the execution of the motor than the cognitive synchronization task. Furthermore, in the same experimental condition, delta and beta band power were increased in the temporo-central ROI. To the best of our knowledge, this is the first time such findings have been observed. The following discussion is consistent with the body of extant neuroscientific knowledge in the field.

First, the delta band showed double synchronization (related to both interoception and motor synchronization), which implies the activation of both frontal and temporo-central regions during the motor compared with the cognitive synchronization task in the focus condition.

As far as the functional significance of the delta band is concerned in this context, the literature is still scant. In former studies exploring Zen and Qi-Gong (Tei et al., 2009) meditators, EEG correlates displayed an augment of frontal delta power during meditative practice, presumably marking the inhibition of cognitive engagement and a higher ability to disengage from the experience. In addition, delta oscillations play a role in homeostasis and autonomic activity (Ako et al., 2003; Knyazev, 2012; Harmony, 2013). However, interestingly, during mental tasks, the increase in delta oscillation was also associated with functional cortical deafferentation, or inhibition of the sensory afferences that interfere with internal concentration (Harmony, 2013). Thus, the presence of this frequency band in frontal sites might be primarily related to the focus on the breath instruction.

Regarding the manifestation of delta in the temporo-central regions, a previous study observed delta oscillations in frontal and central electrode sites as a marker of coordinated and controlled motor activity (Lindenberger et al., 2009). Thus, it might be plausible that the temporo-central presence of delta in this specific condition could be linked to the synergy during the motor synchronization task. Nonetheless, it is interesting to notice that this effect is observed only in the focus on the breath condition (this difference is not observed in the no-focus condition), so this effect could be induced (or made manifest), especially in the case of the interoceptive focus. Given these considerations, it could be concluded that the interoceptive focus might have promoted the activation of this marker that supports motor, rather than cognitive, synchronization (the latter here operationalized with a linguistic synchronization task).

The result observed for the theta band would seem to confirm this trend. The presence of theta was detected here mainly in the anterior frontal area during the motor compared with the cognitive synchronization task in the focus condition. The manifestation of frontal midline theta rhythm was correlated before with mental concentration, task-dependent attention, and a focused meditative state (Kubota et al., 2001). In the study of Tripathi et al. (2022), theta amplitudes and peak frequency increased in the centro-frontal region during the rhythmic breathing period but were marked by sustained low theta waves during the meditation period.

Conversely, in a former EEG study investigating the psychophysiological differences between cognitive and motor tasks, higher spectral power in the theta band at frontal electrodes was found in the cognitive than in the motor task (Ryu et al., 2016), interpreting the increase in theta activity as a correlate of problem-solving. Notwithstanding in this work, the authors exploited two more complex motor and cognitive board games that are different in terms of cognitive demand from those adopted in our study, as well as no synchronization was required.

Instead, in our study, motor synchronization seems to play a fundamental role in the manifestation of EEG patterns. In a study by Zhu et al. (2010), subjects played rhythm games with a keyboard, and their brain waves showed frontal theta power. Furthermore, as shown by Lindenberger et al. (2009) together with delta oscillations, theta band was also observed in frontal electrode sites, which may indicate coordinated and controlled motor activity. Also, theta and delta band synchronization within and between players was enhanced at the frontal and central electrodes during the preparatory tempo setting and at coordinated play onsets when there is a high demand for coordination (Sänger et al., 2012). So, it could be argued that this result for the theta band constitutes evidence of the motor-induced interindividual synergy effect conceived as a real joint action, rather than cognitive synchronization.

On the contrary, the beta band was mainly observed in the temporo-central regions (not frontal ROIs) again during the motor compared with the cognitive synchronization task in the focus condition. Beta-band activity is classically considered as being related to motor functions (Engel and Fries, 2010), and it was also shown to occur in the motor cortex during motor and attention tasks (Khanna and Carmena, 2015). It has also been argued it could have an instrumental role in predictive timing and rhythm during movement synchronization, together with low-frequency oscillations (Arnal, 2012). Previous EEG hyperscanning studies reported the manifestation of beta during behavioral motor synchrony in distinct regions of the brain (Yun et al., 2012), including the centro-parietal areas (Dumas et al., 2010).

Moreover, the beta band increase in the temporo-central region may indicate the sensorimotor system propensity to uphold the *status quo*. In addition, the beta band seems to enable the more effective processing of feedback (e.g., proprioceptive signals), which is necessary for keeping tabs on the state of affairs and readjusting the sensorimotor system (Baker, 2007). In addition, there is proof that the beta band can alter how sensations are processed in the somatosensory cortex (Lalo et al., 2007).

So, it might be possible that the prevalence of beta in the temporo-central (and not frontal) region occurs due to the effect of consonance, intentionality, and awareness showing maximum response in the motor synchronization task, which requires focused attention. Indeed, rhythmic synchronization activities need sensory–motor coordination. However, one alternative explanation of the beta increase could also be related to the normalization procedure adopted in the present study. Future research could better disambiguate this aspect, adopting different methodological approaches.

Taken together, this evidence suggests that the interoceptive focus goes hand in hand with the motor synchronization task, which requires full synergy, without cognitive interference, which the cognitive task could have produced, obscuring the effect of IA. Indeed, different from what was expected and stated in our hypotheses for the cognitive synchronization task, the presence of mainly alpha and theta band wave activities in the temporal and parietal regions during the cognitive synchronization task performance was not detected. No evidence was also observed in the focus condition while the participants were performing the cognitive synchronization task. A possible explanation for this lack of significant results could be due to the nature of the task, which required multiple cognitive processes. In fact, a modified version of the human-to-human alternating speech task, in which the participants had to syllabize with the experimenter for a total of 3 mins, was adopted for the current study. It could be speculated that the mediation of the verbal register has made the request to focus on the breath and synchronize with the speech too complex, and this could have increased the cognitive load in the individuals requiring the activation of a more distributed and differentiated neural network in a non-significant way.

Prospective studies are needed to disentangle the psychophysiological correlates of the effect of IA on cognitive synchronization tasks, adopting more ecological tasks or other neuroscientific methods.

We acknowledge that in our previous fNIRS study, we observed an increase in the right PFC when the explicit focus on the breath was induced during the cognitive task requiring synchronization: however in comparison to these hemodynamic results, the EEG montage was here extended to additional brain regions, and data were, in this case, more precise with reference to the function and the source of the EEG signal. Indeed, cortical oscillations provide useful information to monitor the process beyond localization that could be not linked to the specific processes. Future research in this field would benefit from some methodological improvements, such as (i) the integration of a fNIRS-EEG co-registration over multiple cortical sites; (ii) the implementation of an EEG hyperscanning paradigm, collecting the information of the other partner's brain; and (iii) the integration of more extensive EEG biosignal analysis to parameterize neural power, for instance, the analysis of the oscillatory portion of the spectrum of specific frequency bands (as previously operated by Tripathi et al., 2022).

To conclude, this study suggested two main patterns of EEG frequency band manifestation during the execution of a motor compared with a cognitive synchronization task while a person is focusing the attention on one's breath: significantly higher delta and theta power in the frontal ROI, and delta and beta band power increases in the temporo-central ROI. This evidence indicates that interoception (conceived as the focused attention on the breath) improves the manifestation of EEG brain correlates related to mental concentration, coordinated and controlled motor activity during motor synchronization. It might be plausible that this EEG pattern could have potential benefits on improving motor imitation performance at the behavioral level; however, future studies are needed to confirm this effect. Indeed, this study can be considered as a first attempt to classify the different effects of interoceptive manipulation on cognitive and motor synchronization tasks by comparing them using neurophysiological measures.

## Data availability statement

The raw data supporting the conclusions of this article will be made available by the authors, without undue reservation.

## Ethics statement

The studies involving human participants were reviewed and approved by Department of Psychology, Catholic University of the Sacred Heart, Milan, Italy. The patients/participants provided their written informed consent to participate in this study.

## Author contributions

MB and LA contributed to the conception and design of the study, wrote the first draft, contributed to manuscript revision, read, and approved the submitted version.

## Conflict of interest

The authors declare that the research was conducted in the absence of any commercial or financial relationships that could be construed as a potential conflict of interest.

## Publisher's note

All claims expressed in this article are solely those of the authors and do not necessarily represent those of their affiliated organizations, or those of the publisher, the editors and the reviewers. Any product that may be evaluated in this article, or claim that may be made by its manufacturer, is not guaranteed or endorsed by the publisher.

## References

[B1] Ako M. Kawara T. Uchida S. Miyazaki S. Nishihara K. Mukai J.. (2003). Correlation between electroencephalography and heart rate variability during sleep. Psychiatry Clin. Neurosci. 57, 59–65. 10.1046/j.1440-1819.2003.01080.x 12519456

[B2] Arch J. J. Craske M. G. (2006). Mechanisms of mindfulness: emotion regulation following a focused breathing induction. Behav. Res. Ther. 44, 1849–1858. 10.1016/j.brat.2005.12.007 16460668

[B3] Arnal L. H. (2012). Predicting “when” using the motor system's beta-band oscillations. Front. Hum. Neurosci. 6, 1–3. 10.3389/fnhum.2012.00225 22876228 PMC3410664

[B4] Arnau S. Löffler C. Rummel J. Hagemann D. Wascher E. Schubert A. L. (2020). Inter-trial alpha power indicates mind wandering. Psychophysiology 57, 1–14. 10.1111/psyp.13581 32277853

[B5] Arnold A. J. Winkielman P. Dobkins K. (2019). Interoception and social connection. Front. Psychol. 10, 2589. 10.3389/fpsyg.2019.02589 31849741 PMC6901918

[B6] Baker J. M. Liu N. Cui X. Vrticka P. Saggar M. Hosseini S. M. H.. (2016). Sex differences in neural and behavioral signatures of cooperation revealed by fNIRS hyperscanning. Sci. Rep. 6, 1–11. 10.1038/srep26492 27270754 PMC4897646

[B7] Baker S. N. (2007). Oscillatory interactions between sensorimotor cortex and the periphery. Curr. Opin. Neurobiol. 17, 649–655. 10.1016/j.conb.2008.01.007 18339546 PMC2428102

[B8] Balconi M. Angioletti L. (2021a). Interoception as a social alarm amplification system. What multimethod (EEG-fNIRS) integrated measures can tell us about interoception and empathy for pain? Neuropsychol. Trends 39–64. 10.7358/neur-2021-029-bal1

[B9] Balconi M. Angioletti L. (2021b). One's interoception affects the representation of seeing others' pain: a randomized controlled qEEG study. Pain Res. Manag. 2021, 1–15. 10.1155/2021/5585060 33884043 PMC8041555

[B10] Balconi M. Angioletti L. (2022). Interoceptive attentiveness induces significantly more PFC activation during a synchronized linguistic task compared to a motor task as revealed by functional near-infrared spectroscopy. Brain Sci. 12, 301. 10.3390/brainsci12030301 35326258 PMC8946073

[B11] Balconi M. Molteni E. (2016). Past and future of near-infrared spectroscopy in studies of emotion and social neuroscience. J. Cogn. Psychol. 28, 129–146. 10.1080/20445911.2015.110291927812329

[B12] Balconi M. Vanutelli M. E. (2017). Cooperation and competition with hyperscanning methods: review and future application to emotion domain. Front. Comput. Neurosci. 11, 86. 10.3389/fncom.2017.00086 29033810 PMC5627061

[B13] Baldwin C. L. Roberts D. M. Barragan D. Lee J. D. Lerner N. Higgins J. S. (2017). Detecting and quantifying mind wandering during simulated driving. Front. Hum. Neurosci. 11, 406. 10.3389/fnhum.2017.00406 28848414 PMC5550411

[B14] Braboszcz C. Delorme A. (2011). Lost in thoughts: Neural markers of low alertness during mind wandering. Neuroimage 54, 3040–3047. 10.1016/j.neuroimage.2010.10.008 20946963

[B15] Cheng X. Li X. Hu Y. (2015). Synchronous brain activity during cooperative exchange depends on gender of partner: a fNIRS-based hyperscanning study. Hum. Brain Mapp. 36, 2039–2048. 10.1002/hbm.22754 25691124 PMC6869051

[B16] Colgan D. D. Memmott T. Klee D. Ernst L. Han S. J. Oken B. (2019). A single case design to examine short-term intracranial EGG patterns during focused meditation. Neurosci. Lett. 711, 134441. 10.1016/j.neulet.2019.134441 31430545 PMC6779128

[B17] Compton R. J. Gearinger D. Wild H. (2019). The wandering mind oscillates: EEG alpha power is enhanced during moments of mind-wandering. Cogn. Affect. Behav. Neurosci. 19, 1184–1191. 10.3758/s13415-019-00745-9 31502206

[B18] Cui X. Bryant D. M. Reiss A. L. (2012). NIRS-based hyperscanning reveals increased interpersonal coherence in superior frontal cortex during cooperation. Neuroimage 59, 2430–2437. 10.1016/j.neuroimage.2011.09.003 21933717 PMC3254802

[B19] Descorbeth O. Zhang X. Adam Noah J. Hirsch J. (2020). Neural processes for live pro-social dialogue between dyads with socioeconomic disparity. Soc. Cogn. Affect. Neurosci. 15, 875–887. 10.1093/scan/nsaa120 32879986 PMC7543936

[B20] Dikker S. Wan L. Davidesco I. Kaggen L. Oostrik M. McClintock J.. (2017). Brain-to-brain synchrony tracks real-world dynamic group interactions in the classroom. Curr. Biol. 27, 1375–1380. 10.1016/j.cub.2017.04.002 28457867

[B21] Dumas G. Nadel J. Soussignan R. Martinerie J. Garnero L. (2010). Inter-brain synchronization during social interaction. PLoS ONE 5, e12166. 10.1371/journal.pone.0012166 20808907 PMC2923151

[B22] Engel A. K. Fries P. (2010). Beta-band oscillations-signalling the status quo? Curr. Opin. Neurobiol. 20, 156–165. 10.1016/j.conb.2010.02.015 20359884

[B23] Farb N. A. S. Segal Z. V. Anderson A. K. (2013). Mindfulness meditation training alters cortical representations of interoceptive attention. Soc. Cogn. Affect. Neurosci. 8, 15–26. 10.1093/scan/nss066 22689216 PMC3541492

[B24] Funane T. Kiguchi M. Atsumori H. Sato H. Kubota K. Koizumi H. (2011). Synchronous activity of two people's prefrontal cortices during a cooperative task measured by simultaneous near-infrared spectroscopy. J. Biomed. Opt. 16, 077011. 10.1117/1.3602853 21806291

[B25] Grossman P. (2011). Defining mindfulness by how poorly i think i pay attention during everyday awareness and other intractable problems for psychology's (re)invention of mindfulness: Comment on Brown et al. (2011). Psychol. Assess. 23, 1034–1040. 10.1037/a0022713 22122674

[B26] Harmony T. (2013). The functional significance of delta oscillations in cognitive processing. Front. Integr. Neurosci. 7, 83. 10.3389/fnint.2013.00083 24367301 PMC3851789

[B27] Hirsch J. Noah J. A. Zhang X. Dravida S. Ono Y. (2018). A cross-brain neural mechanism for human-to-human verbal communication. Soc. Cogn. Affect. Neurosci. 13, 907–920. 10.1093/scan/nsy070 30137601 PMC6137318

[B28] Holper L. Scholkmann F. Wolf M. (2012). Between-brain connectivity during imitation measured by fNIRS. Neuroimage 63, 212–222. 10.1016/j.neuroimage.2012.06.028 22732563

[B29] Jasper H. H. (1958). The ten-twenty electrode system of International Federation EEG. Electroencephalogr. Clin. Neurophysiol. 10, 371–375. 10590970

[B30] Jiang J. Dai B. Peng D. Zhu C. Liu L. Lu C. (2012). Neural synchronization during face-to-face communication. J. Neurosci. 32, 16064–16069. 10.1523/JNEUROSCI.2926-12.2012 23136442 PMC6621612

[B31] Kawasaki M. Yamada Y. Ushiku Y. Miyauchi E. Yamaguchi Y. (2013). Inter-brain synchronization during coordination of speech rhythm in human-to-human social interaction. Sci. Rep. 3, 1692. 10.1038/srep01692 23603749 PMC3631767

[B32] Kelsen B. A. Sumich A. Kasabov N. Liang S. H. Y. Wang G. Y. (2022). What has social neuroscience learned from hyperscanning studies of spoken communication? A systematic review. Neurosci. Biobehav. Rev. 132, 1249–1262. 10.1016/j.neubiorev.2020.09.008 33022298

[B33] Khanna P. Carmena J. M. (2015). Neural oscillations: beta band activity across motor networks. Curr. Opin. Neurobiol. 32, 60–67. 10.1016/j.conb.2014.11.010 25528615

[B34] Knyazev G. G. (2012). EEG delta oscillations as a correlate of basic homeostatic and motivational processes. Neurosci. Biobehav. Rev. 36, 677–695. 10.1016/j.neubiorev.2011.10.002 22020231

[B35] Kubota Y. Sato W. Toichi M. Murai T. Okada T. Hayashi A.. (2001). Frontal midline theta rhythm is correlated with cardiac autonomic activities during the performance of an attention demanding meditation procedure. Cogn. Brain Res. 11, 281–287. 10.1016/S0926-6410(00)00086-0 11275489

[B36] Lalo E. Gilbertson T. Doyle L. Di Lazzaro V. Cioni B. Brown P. (2007). Phasic increases in cortical beta activity are associated with alterations in sensory processing in the human. Exp. Brain Res. 177, 137–145. 10.1007/s00221-006-0655-8 16972074

[B37] Lindenberger U. Li S. C. Gruber W. Müller V. (2009). Brains swinging in concert: Cortical phase synchronization while playing guitar. BMC Neurosci. 10, 93–96. 10.1186/1471-2202-10-22 19292892 PMC2662862

[B38] Liu T. Saito G. Lin C. Saito H. (2017). Inter-brain network underlying turn-based cooperation and competition: a hyperscanning study using near-infrared spectroscopy. Sci. Rep. 7, 1–12. 10.1038/s41598-017-09226-w 28819162 PMC5561070

[B39] Lomas T. Ivtzan I. Fu C. H. Y. (2015). A systematic review of the neurophysiology of mindfulness on EEG oscillations. Neurosci. Biobehav. Rev. 57, 401–410. 10.1016/j.neubiorev.2015.09.018 26441373

[B40] Montague P. R. Berns G. S. Cohen J. D. McClure S. M. Pagnoni G. Dhamala M.. (2002). Hyperscanning: simultaneous fMRI during linked social interactions. Neuroimage 16, 1159–1164. 10.1006/nimg.2002.1150 12202103

[B41] Pan Y. Cheng X. Zhang Z. Li X. Hu Y. (2017). Cooperation in lovers: an fNIRS-based hyperscanning study. Hum. Brain Mapp. 38, 831–841. 10.1002/hbm.23421 27699945 PMC6867051

[B42] Pérez A. Carreiras M. Duñabeitia J. A. (2017). Brain-to-brain entrainment: EEG interbrain synchronization while speaking and listening. Sci. Rep. 7, 1–12. 10.1038/s41598-017-04464-4 28646190 PMC5482847

[B43] Ryu K. Choi Y. Kim J. Kim Y. Chio S. (2016). Differential frontal theta activity during cognitive and motor tasks. J. Integr. Neurosci. 15, 295–303. 10.1142/S0219635216500199 27550366

[B44] Sänger J. Müller V. Lindenberger U. (2012). Intra- and interbrain synchronization and network properties when playing guitar in duets. Front. Hum. Neurosci. 6, 312. 10.3389/fnhum.2012.00312 23226120 PMC3509332

[B45] Scholkmann F. Holper L. Wolf U. Wolf M. (2013). A new methodical approach in neuroscience: assessing inter-personal brain coupling using functional near-infrared imaging (fNIRI) hyperscanning. Front. Hum. Neurosci. 7, 813. 10.3389/fnhum.2013.00813 24348362 PMC3841755

[B46] Schulz S. M. (2016). Neural correlates of heart-focused interoception: A functional magnetic resonance imaging meta-analysis. Philos. Trans. R. Soc. B Biol. Sci. 371, 20160018. 10.1098/rstb.2016.0018 28080975 PMC5062106

[B47] Tei S. Faber P. L. Lehmann D. Tsujiuchi T. Kumano H. Pascual-Marqui R. D.. (2009). Meditators and non-meditators: EEG source imaging during resting. Brain Topogr. 22, 158–165. 10.1007/s10548-009-0107-4 19653090

[B48] Tripathi V. Bhasker L. Kharya C. Bhatia M. Kochupillai V. (2022). Electroencephalographic dynamics of rhythmic breath-based meditation. bioRxiv, 2022.03.09.483685. 10.1101/2022.03.09.483685v2.abstract

[B49] Tsakiris M. De Preester H. (2018). The Interoceptive Mind: From Homeostasis to Awareness. Oxford: Oxford University Press.

[B50] van Son D. De Blasio F. M. Fogarty J. S. Angelidis A. Barry R. J. Putman P. (2019). Frontal EEG theta/beta ratio during mind wandering episodes. Biol. Psychol. 140, 19–27. 10.1016/j.biopsycho.2018.11.003 30458199

[B51] Villena-González M. Moënne-Loccoz C. Lagos R. A. Alliende L. M. Billeke P. Aboitiz F.. (2017). Attending to the heart is associated with posterior alpha band increase and a reduction in sensitivity to concurrent visual stimuli. Psychophysiology 54, 1483–1497. 10.1111/psyp.12894 28560781

[B52] Weng H. Y. Feldman J. L. Leggio L. Napadow V. Park J. Price C. J. (2021). Interventions and manipulations of interoception. Trends Neurosci. 44, 52–62. 10.1016/j.tins.2020.09.010 33378657 PMC7805576

[B53] Yun K. Watanabe K. Shimojo S. (2012). Interpersonal body and neural synchronization as a marker of implicit social interaction. Sci. Rep. 2, 959. 10.1038/srep00959 23233878 PMC3518815

[B54] Zhang Y. Meng T. Hou Y. Pan Y. Hu Y. (2018). Interpersonal brain synchronization associated with working alliance during psychological counseling. Psychiatry Res. Neuroimaging 282, 103–109. 10.1016/j.pscychresns.2018.09.007 30292535

[B55] Zhu F. F. Maxwell J. P. Hu Y. Zhang Z. G. Lam W. K. Poolton J. M.. (2010). EEG activity during the verbal-cognitive stage of motor skill acquisition. Biol. Psychol. 84, 221–227. 10.1016/j.biopsycho.2010.01.015 20117168

